# Facile Coaxial Electrospinning Synthesis of Polyacrylonitrile/Cellulose Acetate Nanofiber Membrane for Oil–Water Separations

**DOI:** 10.3390/polym15234594

**Published:** 2023-11-30

**Authors:** Maha Mohammad AL-Rajabi, Ismail W. Almanassra, Abdelrahman K. A. Khalil, Muataz Ali Atieh, Tahar Laoui, Khalil Abdelrazek Khalil

**Affiliations:** 1 Research Institute of Sciences and Engineering, University of Sharjah, Sharjah 27272, United Arab Emirates; maha.alrajabi@unimap.edu.my (M.M.A.-R.); ialmanassra@sharjah.ac.ae (I.W.A.); abdelrahman.khalil@sharjah.ac.ae (A.K.A.K.); mhussien@sharjah.ac.ae (M.A.A.); tlaoui@sharjah.ac.ae (T.L.); 2 Faculty of Chemical Engineering & Technology, Universiti Malaysia Perlis, UniMAP, Arau 02600, Perlis, Malaysia; 3 Centre of Excellence for Biomass Utilization, Universiti Malaysia Perlis, UniMAP, Arau 02600, Perlis, Malaysia; 4 Chemical and Water Desalination Engineering Program, College of Engineering, University of Sharjah, Sharjah 27272, United Arab Emirates; 5 Department of Mechanical and Nuclear Engineering, College of Engineering, University of Sharjah, Sharjah 27272, United Arab Emirates

**Keywords:** nanofibers, membrane, electrospinning, oil–water separation, hydrophilic–oleophobic

## Abstract

Oil-contaminated water and industrial oily wastewater discharges have adversely affected aquatic ecosystems and human safety. Membrane separation technology offers a promising solution for effective oil–water separation. Thus, a membrane with high surface area, hydrophilic–oleophobic properties, and stability is a promising candidate. Electrospinning, a straightforward and efficient process, produces highly porous polymer-based membranes with a vast surface area and stability. The main objective of this study is to produce hydrophilic–oleophobic polyacrylonitrile (PAN) and cellulose acetate (CA) nanofibers using core–shell electrospinning. Incorporating CA into the shell of the nanofibers enhances the wettability. The core PAN polymer improves the electrospinning process and contributes to the hydrophilicity–oleophobicity of the produced nanofibers. The PAN/CA nanofibers were characterized by Fourier transform infrared spectroscopy, field emission scanning electron microscopy, X-ray diffraction, and surface-wetting behavior. The resulting PAN/cellulose nanofibers exhibited significantly improved surface-wetting properties, demonstrating super-hydrophilicity and underwater superoleophobicity, making them a promising choice for oil–water separation. Various oils, including gasoline, diesel, toluene, xylene, and benzene, were employed in the preparation of oil–water mixture solutions. The utilization of PAN/CA nanofibers as a substrate proved to be highly efficient, confirming exceptional separation efficiency, remarkable stability, and prolonged durability. The current work introduces an innovative single-step fabrication method of composite nanofibers, specially designed for efficient oil–water separation. This technology exhibits significant promise for deployment in challenging situations, offering excellent reusability and a remarkable separation efficiency of nearly 99.9%.

## 1. Introduction

In the current vast, growing industrial age, spoilage of oil-contaminated water and industrial oily wastewater discharges have adversely affected the aquatic ecosystem and human safety [1]. According to the International Tanker Owners Pollution Federation, more than 1800 significant incidents involving oil tankers occurred between 1970 and 2015, resulting in the release of roughly 5.72 million metric tons of oil [2]. Thus, effective oil–water separation has become increasingly crucial for the sustainable development of the ecosystem. Various traditional techniques have been employed in the past for the separation of oil and water, including processes like air flotation [3], coagulation [4], and controlled burning [5]. Nevertheless, these approaches face limitations due to their low separation efficiency, high energy consumption, the generation of secondary pollutants, and time-consuming separation techniques [2]. Hence, there is an urgent need to develop economically efficient techniques for effectively separating oil–water mixtures. Therefore, an urgent demand is placed on developing cost-effective methods to separate oil–water mixtures efficiently. Among the available oil–water separation methods, membrane-based separation technology is gaining importance due to its simple operation, high operational efficiency, and environmentally friendly attributes.

Membrane separation technology represents a promising approach for oil–water separation. The innovative membrane materials can be classified into two main types for oil–water separation: hydrophobic–oleophilic and hydrophilic–oleophobic membranes. Particularly, hydrophilic–oleophobic materials are widely used as the optimal choice for effective oil–water separation due to limitations in hydrophobic–oleophilic membranes [6]. The latter type permits oil to pass through the membrane while repelling water. This mechanism results in oil blockage of the pore, which adversely affects membrane performance and restricts its reusability. Moreover, these membranes when exposed to oily water become brittle, limiting their multiple applications [7]. The hydrophilic–oleophobic variant has garnered significant attention from researchers for its potential to address fouling concerns. It combines high hydrophilicity with a hierarchical surface structure that enables water passage through the membrane. This unique combination results in high water flux while preventing oil from adhering to the fiber surface [7].

Various techniques are employed to fabricate these membranes, including phase inversion [8], coating [9], sol–gel [10], and electrospinning [7] methods. Of all these methods, electrospinning stands as a straightforward and efficient process for producing polymer-based membranes with high porosity, desirable surface properties, and exceptional permeation rates [11]. Electrospinning technology enables the fabrication of nanofibers using a diverse range of materials, either natural or synthetic polymers, such as poly(vinylidene fluoride) (PVDF) [12], polyvinyl alcohol [13], nylon 6 [14], polyethylene glycol [15], polyacrylonitrile [15], and cellulose acetate (CA) [2]. CA is considered the most important organic ester of cellulose, an abundant polymer in nature [16,17,18,19]. CA is extensively used in many applications, including plastics, coatings, and fibers, due to its advantages, including non-toxicity and low cost compared to other polymers [20].

Similarly, CA nanofibers showed unique properties, including biodegradability, thermal stability, and good chemical resistance [21]. However, pure electrospun CA nanofibers often exhibit poor mechanical strength and unsatisfactory microstructure due to inadequate fiber–fiber interconnection [22]. Consequently, enhancing the mechanical properties and morphology of CA nanofibers is essential. One effective approach is to introduce a polymer with good mechanical properties into a CA nanofiber matrix, resulting in the production of high-performance composite nanofibers. Polyacrylonitrile (PAN) is recognized for its commercial significance with many attractive and desirable properties including high thermal stability and resilience to environmental factors and robust mechanical strength [2]. This elevated mechanical strength is attributed to the nitrile group in PAN which has a high cohesive energy [22]. Additionally, PAN nanofibers are amphiphilic materials with robust mechanical properties. To combine the properties of the two polymers, many researchers attempt to synthesize PAN/CA nanofibers.

Wang et al. (2020) produced electrospun PAN/CA nanofibrous membranes with controllable spatial structures through a multi-fluid-mixing electrospinning process utilizing a multi-spinneret. The research team proposed that the composite nanofibers composed of 75% CA:25% PAN possess promising application in the field of membrane separation, primarily owing to their desirable combination of hydrophobicity and mechanical properties. However, no application was studied in this research [22]. Karki et al. (2019) produced PAN/CA composite nanofibers by stacking fiber layers one over another using a hot roller. The produced fibers showed hydrophobic properties, and surface modification was performed to render it hydrophilic. The transformation of PAN/CA composite fibers from hydrophobic fibers to hydrophilic fibers was achieved through a deacetylation process, which involved alkaline hydrolysis using sodium hydroxide and an aqueous ethanolic solution. The resulting composite fibers exhibited both a high water permeation flux and excellent recyclability, making it a promising candidate for separating of oil–water solutions [2].

However, studies in the literature that combine PAN/CA to produce nanofibers have focused on the synthesis of nanofibers using each polymer separately and then combining the produced nanofibers and, thus, requiring surface modification [2] or combining these polymers during electrospinning where multi-fluid-mixing electrospinning processes (multi-spinneret) are needed [22]. Combining CA and PAN to produce PAN/CA nanofibers using simple one-step, single spinneret, and improved properties is required. This can be achieved using coaxial (core and shell) electrospinning. Coaxial electrospinning is an effective technique for producing composite materials to use for specific applications [21]. Coaxial electrospinning is a technique based on a con-current co-spinning of two polymeric liquids, involving “core and shell” electrospinning on two needles from two syringe pumps, designed in a coaxial mode [23]. This is a modified version of electrospinning that enables nanofiber fabrication with core–shell morphology [23]. Producing novel hydrophilic–oleophobic PAN/CA nanofibers using core–shell electrospinning for oil–water separation applications is promising. The CA molecular chain has both hydrophobic acetyl groups and hydrophilic hydroxyl groups [22]. Adding CA to the shell of the nanofibers is expected to expose the hydrophilic hydroxyl groups of the molecular chain of CA to the surface, thus improving the wettability behavior of the produced nanofibers. The core PAN polymer is expected to enhance the electrospinnability of the nanofibers, which is considered a problem in CA polymer alone, and support the hydrophilicity–oleophobicity of the produced nanofibers constituting the best choice for oil–water separation. The main objective of the current study is to produce hydrophilic–oleophobic PAN/CA nanofibers using core–shell electrospinning for oil–water separation.

## 2. Materials and Methods

### 2.1. Materials

Polyacrylonitrile (PAN) (molecular weight (MW): 230 kDa) in the form of a copolymer with 99.5% acrylonitrile and 0.5% methyl acrylate and cellulose acetate (CA) (average MW: 50 kDa) were used in this study. PAN and CA were supplied by Sigma–Aldrich, Hamburg, Germany. Acetone and dimethylformamide (DMF) were purchased from Merck Millipore, Darmstadt, Germany. Diesel and gasoline were obtained from a local gas station (Emirates Gas Station, Sharjah, United Arab Emirates). Additionally, xylene (C_8_H_10_) with a purity of 98.5%, benzene (C_6_H_6_) with a purity of 99.7%, and toluene (C_7_H_8_) with a purity of 99.5% were procured from SDFCL in Mumbai, India.

### 2.2. Synthesis of PAN/CA Nanofibers

**Figure 1:**
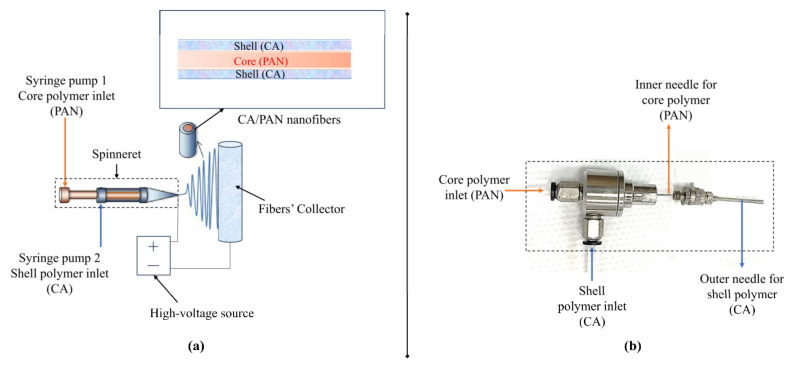
A schematic diagram of (**a**) core–shell electrospinning of PAN/CA nanofibers and (**b**) a photograph of the spinneret.

A 10 wt% solution of CA was prepared by dissolving a specific amount of CA in a DMF/acetone (1/1) mixture at 80 °C for 12 h. A 5 wt% PAN solution in DMF was prepared by stirring for 24 h at 50 °C. The fabrication of PAN/CA nanofibers was carried out using an electrospinning device (Nanospinner, Inovenso nanospinner, Inovenso Inc., Boston, MA, USA). In this process, the 5 wt% PAN solution and the 10 wt% CA solution were loaded into separate 10 mL syringes, positioned in their respective 1 and 2 positions within the syringe pump device, as illustrated in Figure 1. PAN and CA solutions inside the syringe were injected through a plastic tube as core and shell solutions through a coaxial spinneret (Figure 1b). The coaxial spinneret had an inner needle size of 20 G (inner and outer diameters of 0.603 mm and 0.908 mm, respectively) and an outer needle size of 14 G (inner and outer diameters of 1.600 mm and 2.108 mm, respectively). PAN/CA nanofibers were prepared using five different core-to-shell flowrate ratios, as listed in Table 1. A constant voltage of 11 kV, generated by a power supply, was applied between the core–shell spinneret and the collector, which was covered with aluminum foil. The collector’s rotational speed was fixed at 150 rpm, and it was positioned 12 cm away from the needle tip. The electrospinning process was performed at room temperature, ranging from 24–26 °C, with a relative humidity fluctuating between 50–60%. Subsequently, to produce robust and dimensionally uniform membranes, the PAN/CA nanofibers were hot-pressed for 2 min at 140 °C using a hand-held electric iron with a pressure of approximately 3 kPa [7].

### 2.3. Characterization of PAN/CA Nanofibers

#### 2.3.1. Functional Groups

To confirm the functional groups on the PAN/CA nanofibers, Fourier transform infrared spectroscopy (FTIR) was conducting using a JASCO FTIR-6300 (JASCO, Tokyo, Japan). This analysis employed the KBr mode, involving 32 scans within a spectral range extending from 500 cm^−1^ to 4000 cm^−1^.

#### 2.3.2. Surface Morphology and Structure

The observation of the top surface morphology of the PAN/CA nanofibers was conducted through a field emission scanning electron microscope (FESEM), Apreo, Thermo Fisher Scientific, Waltham, MA, USA. To facilitate imaging, a thin layer of gold was coated onto the PAN/CA nanofibers using a vacuum sputter coater. The top surface morphology was observed at both 10 k× and 30 k× magnifications. The average fiber diameter of the PAN/CA nanofibers was determined using ImageJ software (Version 1.53c, Java 1.8.0/172, NIH, Bethesda, MD, USA). The results are presented as average fiber diameter ± standard deviation, which was calculated using the STDEV formula in Excel.

#### 2.3.3. Crystallinity

The crystalline properties of PAN/CA nanofibers were evaluated using X-ray diffraction (XRD) employing a D8 Advance X-ray diffractometer (Bruker AXS, Karlsruhe, Germany) equipped with a CuKα radiation source at a wavelength of 1.5406 Å. The XRD patterns were obtained withing a 2θ scan range from 10° to 70°, utilizing a step size of 0.025° and an exposure rate of 0.1 s/step.

#### 2.3.4. Contact Angle

The measurement of water contact angle and underwater oil contact angle for each PAN/CA nanofiber was conducted using a contact angle meter (Ramé-hart Instrument Co., Succasunna, NJ, USA) accompanied by Drop Shape Analysis Software (version no. 3.54.164.0). The contact angle was measured at three different spots on the PAN/CA nanofibers’ surface to minimize analytical errors.

### 2.4. Performance of PAN/CA Nanofibers

#### Oil–Water Mixture Separation

**Figure 2:**
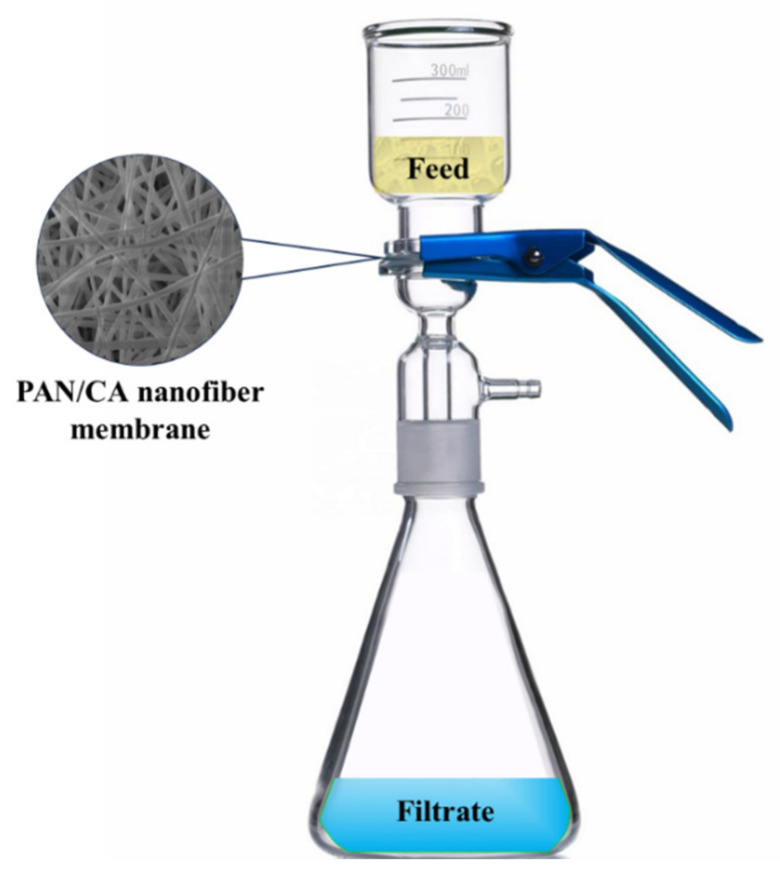
Diagram depicting the oil–water separation system.

A 3:1 ratio mixture of 75 mL deionized water and 25 mL targeted oil was prepared for membrane efficiency evaluation. After 24 h of initial agitation, the emulsion was further stabilized by vigorous stirring at 1500 rpm for 30 min. This mechanical agitation, combined with the inherent hydrophilic–oleophobic properties of PAN/CA nanofibers, ensured the formation of a stable and homogeneous oil–water emulsion. Filtration experiments started immediately after stirring, utilizing a gravity-driven system with a 4 cm diameter filtration circle, as depicted in Figure 2. Each separation trial, assessing membrane reusability, was conducted over a minimum of three cycles, involving flash rinsing with water only. To confirm the durability and reusability of the membranes, 3 consecutive filtration cycles were conducted for gasoline, benzene, and toluene while 5 filtration cycles were conducted for diesel and xylene. All filtration experiments were conducted at room temperature.

The membrane’s permeation flux (F) in L/m^2^/h (LMH) was determined utilizing Equation (1), where ”m” represents the mass of the water permeated through the membrane (g), ”A” denotes the effective area of the membrane (12.57 × 10^−4^ m^2^), ρ stands for the density of the water at room temperature (997.8 g/L), and ”t” signifies the duration of the separation test.

(1) $$F=\frac{m}{A\times \rho \times t}$$

The normalized flux was calculated according to Equation (2), where $J_{2\;\mathrm{or}\;3}$(LMH) is the permeate flux of the second or third cycle, and $J_{1}$(LMH) represents the permeate flux of the first filtration cycle.

(2) $$\mathrm{Normalized}\;\mathrm{flux}=\frac{J_{2\;\mathrm{or}\;3}}{J_{1}}$$

The removal efficiency was calculated according to Equation (3), where $T_{f}$ and $T_{p}$ are the turbidity of the feed and permeate in (FNU), respectively.

(3) $$\%\;\mathrm{Removal}\;\mathrm{efficiency}\;=\frac{T_{f}-T_{p}}{T_{f}}\times 100\%$$

Following the separation experiment, the permeates were analyzed for their oil content, employing a turbidity meter model MI415 PRO manufactured by Milwaukee in Romania. These data were then paired with the initial oil concentration of the feed emulsion. This comparative analysis enabled the calculation of both the removal efficiency measurements and the membrane’s separation efficiency, providing a comprehensive assessment of the membrane’s performance in oil–water separation.

## 3. Results and Discussion

### 3.1. Characterization of PAN/CA Nanofibers

#### 3.1.1. Functional Groups

**Figure 3:**
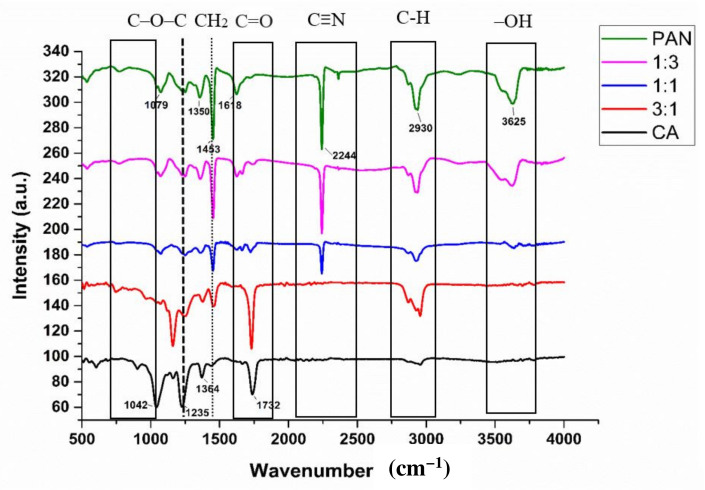
FTIR spectra of PAN/CA nanofibers.

The FTIR spectra of PAN/CA nanofibers are presented in Figure 3. In pure CA nanofibers, four distinct peaks were identified. The peak observed at 1732 cm^−1^ is associated with the stretching of the C=O ester carbonyl [24,25], that at 1364 cm^−1^ is attributed to the bending vibration of the C–H bond in the acetyl groups [25], that a 1235 cm^−1^ arises from stretching vibrations of the C–O–C bond in acetyl groups [25,26], and the peak at 1042 cm^−1^ is characteristic of the C–O–C pyranose ring’s skeletal vibration [25]. CA contains both acetyl groups and hydroxyl groups. The presence of acetyl-group peaks in the FTIR spectra indicates the hydrophobic nature of pure CA nanofibers. Acetyl groups are known for their hydrophobic properties. This information is essential in studying the wettability of the nanofibers, considering their application in separation. Notably, the absence of a hydroxyl-group peak in the FTIR spectra of pure CA nanofibers suggests that the hydroxyl groups are not exposed on the surface. Hydroxyl groups are associated with hydrophilic characteristics; limited surface exposure of hydroxyl groups may contribute to the hydrophobic nature of the pure CA nanofibers.

Conversely, PAN within the PAN/CA nanofibers presents a simple chemical composition, characterized by a C–C backbone and an abundance of C≡N groups. The FTIR spectra of pure PAN nanofibers revealed distinctive peaks. Firstly, a sharp and narrow peak at 2244 cm^−1^ was ascribed to the stretching vibration of the C≡N group [22], while a peak at 1618 cm^−1^ was attributed to C=N [27]. Additionally, the stretching vibration peak at 1350 cm^−1^ was attributed to C–N bonds [28]. A broad peak at 3625 cm^−1^ belonged to the stretching vibration of –OH [29]. The strong peak at 1453 cm^−1^ was due to the bending vibrations of the CH_2_ groups of PAN nanofibers, while peaks at 2930 cm^−1^ and 1079 cm^−1^ belonged to C–H stretching vibrations [29] and C–H in-plane bending vibrations [30], respectively.

In Figure 3, PAN/CA nanofibers exhibit similar absorption bands to both CA and PAN nanofibers, but some peaks attributed to CA are weakened, while those related to PAN are strengthened. This change is due to PAN’s presence in the nanofibers’ core. The peaks at 1732 cm^−1^, 1235 cm^−1^, and 1042 cm^−1^ corresponding to the stretching of C=O ester carbonyl, stretching vibrations of C–O–C bonds in acetyl groups, and the C–O–C pyranose ring’s skeletal vibration, respectively, were weakened in PAN/CA nanofibers. On the other hand, the peaks belonging to PAN were strengthened in the PAN/CA nanofibers, found at 3625 cm^−1^, 2930 cm^−1^, and 2244 cm^−1^ and belonging to stretching vibrations of –OH, C–H stretching vibrations, and stretching vibrations of C≡N, respectively. 

The differences between the spectra of CA, PAN, and PAN/CA nanofibers indicated that the electrospun nanofibers consist of both CA and PAN, originally present in the two polymer solutions. There is no evidence of a chemical bond formed during the electrospinning process, suggesting that the CA and PAN nanofibers are physically connected.

#### 3.1.2. Structure and Surface Morphology

**Figure 4:**
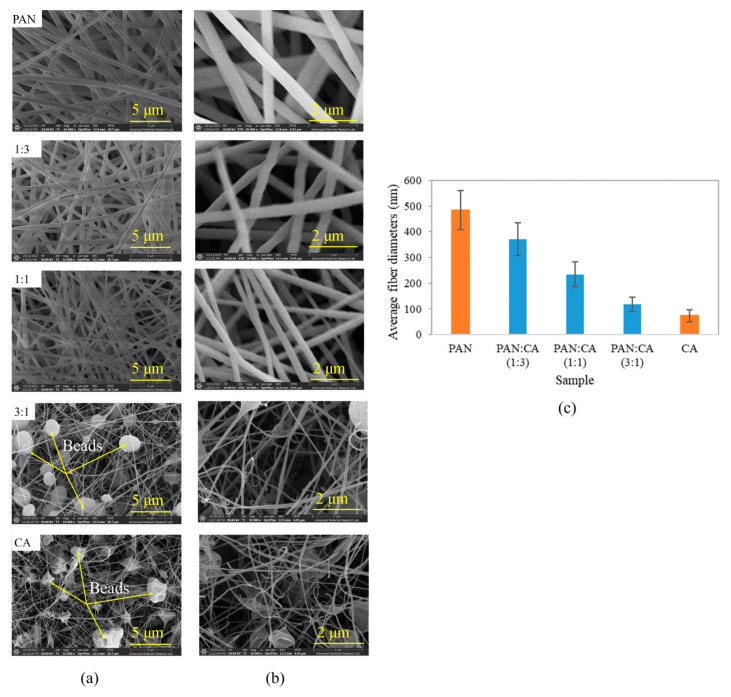
FESEM micrographs of PAN/CA nanofibers at magnifications of (**a**) 10 k× and (**b**) 30 k×, and (**c**) average fiber diameters of PAN/CA nanofibers.

Figure 4 illustrates the surface morphology of PAN/CA nanofibers. Generally, the surfaces of PAN and 1:3 and 1:1 PAN/CA nanofibers were smooth without beads, and each nanofiber was arranged individually to form a weblike structure. However, 3:1 PAN/CA and CA nanofibers showed beaded nanofibers. The presence of beaded nanofibers in pure CA nanofibers can be explained by the low viscosity of the CA solution (100–200 cP) [31], resulting in low levels of polymer chain entanglement and a higher number of beads in nanofibers obtained instead of smooth fibers. In addition to low viscosity, the surface tension of the CA solution might cause the solution to break up into droplets when the solution is stretched, resulting in beaded nanofibers [32]. However, beads on 3:1 PAN/CA nanofibers could be due to high core-to-shell flow ratios. As the core flow rate increased, more PAN solution was ejected from the needle simultaneously. Excessive solution volume leads to bead formation, a phenomenon most likely induced by increased surface tension [33]. Moreover, when the core solution’s flow rate is excessively high (3:1 PAN:CA), the Taylor cone’s size expands beyond optimal dimensions. Consequently, the viscosity drag force exerted by the shell solution becomes insufficient to encapsulate the core entirely. This leads to a loss of the cones’ characteristic shape due to intermixing of both internal and external fluids. To ensure the consistent and uniform encapsulation of the core within the shell, it is important to maintain a lower output rate for the core solution compared to that of the shell solution [34].

The average fiber diameter of the PAN/CA nanofibers is shown in Figure 4c. As shown in Figure 4c, the diameter of all nanofibers was between 73–484 nm, which is less than 1000 nm, making them nanofibers [35]. Additionally, the average diameter of the nanofibers changed with changing core-to-shell flow ratios in the following order: pure PAN > 1:3 (PAN:CA) > 1:1 (PAN:CA) > 3:1 (PAN:CA) > pure CA. Increasing the core-to-shell flow reduced the average diameter of the nanofibers. This can be explained by the increasing flow rate of the core solution, which may lead to effectively reducing the amount of shell polymer surrounding the core material. This reduction in shell polymer material contributes to the decrease in overall nanofiber diameter.

#### 3.1.3. Structure of Membranes

**Figure 5:**
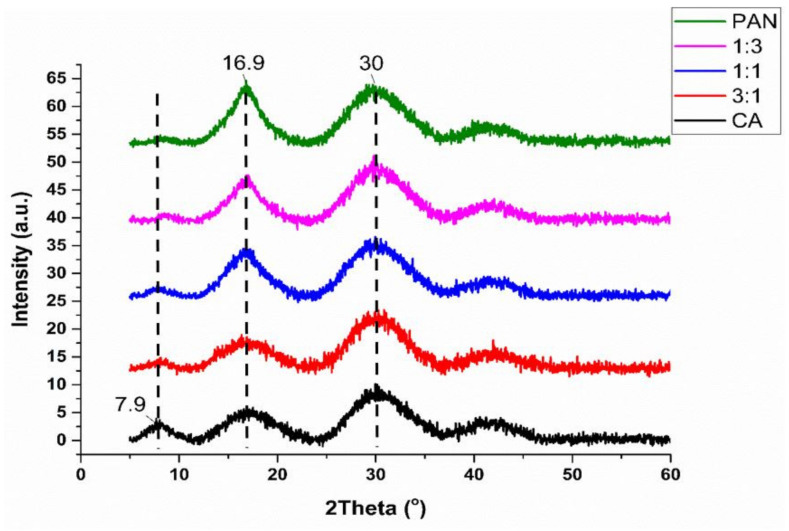
XRD micrographs of PAN/CA nanofibers.

The XRD pattern of PAN/CA nanofibers is presented in Figure 5. As depicted by the XRD diffraction pattern in Figure 5, pure PAN nanofibers exhibit a strong peak at 16.9° and another diffraction peak at 30°. These peaks reflect the ordered crystal structure in PAN linear macromolecules. Specifically, the peak at 16.9° corresponds to the crystalline peak (100) plane of the hexagonal unit of the C≡N groups [36]. In contrast, the 30° peak corresponds to the (110) crystal plane, indicating the distance between the closely parallel molecular segments [37]. It is worth mentioning that the structure of the PAN copolymer has three primary peaks observed in diffraction patterns at ~17°, ~26.0–27.5°, and ~29.5° [38].

In contrast, CA nanofibers exhibit a primary peak around 7.9°, the principal characteristic peak of semicrystalline acetylated cellulose [39]. The position of this peak suggests disorder generation during electrospinning of CA. Additionally, a peak at 16.9° is attributed to reflections from (101) crystalline planes of the cellulose biopolymer, consistent with the expected semicrystalline nature of CA. Santos-Sauceda et al. also reported comparable findings concerning the crystalline structure of CA fibers [40].

The XRD pattern of PAN/CA nanofibers indicates the presence of both CA and PAN peaks. However, the peak at 16.9° is less intense compared to PAN and more intense compared to CA, indicating a higher crystallinity than CA nanofibers, likely due to PAN’s inclusion in the nanofibers’ core. Notably, the intensity of the semicrystalline peak around 7.9° decreases and almost disappears in PAN/CA nanofibers, again suggesting the influence of PAN in the core on the XRD pattern.

#### 3.1.4. Contact Angle

**Figure 6:**
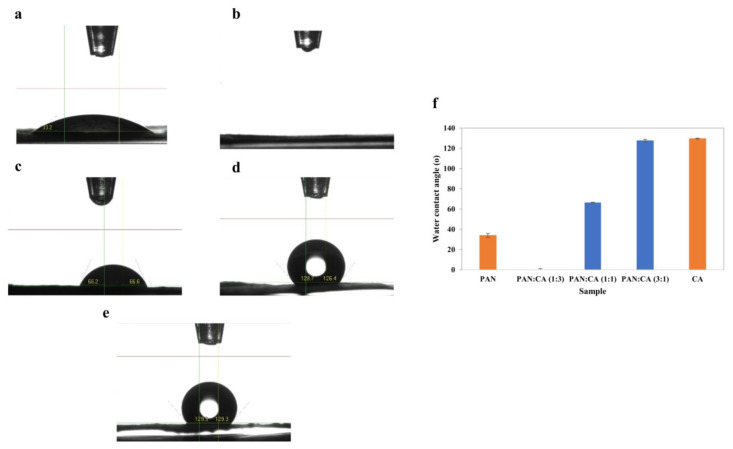
Images of the water droplets on (**a**) PAN (**b**) 1:3 (**c**) 1:1 (**d**) 3:1 and (**e**) CA nanofibers and (**f**) water contact angle of PAN/CA nanofibers.

Surface hydrophilicity–hydrophobicity is an essential factor determining the applications of PAN/CA nanofibers. Figure 6 shows images of water droplets on PAN/CA nanofibers and the water contact angle of PAN/CA nanofibers. Figure 6a shows that nanofibers containing pure PAN were highly hydrophilic with a water contact angle of 33.9°. This hydrophilicity of PAN nanofibers is mainly attributed to the presence of –OH groups within PAN nanofibers, evident in the significantly high intensity of –OH functional group as observed in the corresponding FTIR spectra (Figure 3). In contrast, the water contact angle of pure CA nanofibers was approximately 129.6° (Figure 6e). The molecular structure of CA comprises both hydrophilic hydroxyl groups and hydrophobic groups, with the hydrophilic groups not being exposed on the surface of pure CA nanofibers [22].

Conversely, in the case of pure CA nanofibers, the hydrophobic acetyl group was visibly exposed on the surface. This exposure is validated by the higher intensities of the C=O and C–O–C functional groups in the FTIR spectra (Figure 3). The presence of a hydrophobic acetyl group is responsible for the reduced hydrophilicity observed in pure CA nanofibers. Interestingly, the water droplet was immediately spread and permeated in the surface of PAN/CA nanofibers with a 1:3 PAN:CA ratio showing super-hydrophilic properties. This can be explained by including CA in the shell of the nanofibers, exposing the hydrophilic hydroxyl groups of the molecular chain of CA to the surface, thus improving the wettability behavior of the produced nanofibers. This also can explain the increase in the water contact angle from 0 to 127.5° with an increase in the PAN:CA ratio from 1:3 to 3:1 in PAN/CA nanofibers (Figure 6).

**Figure 7:**
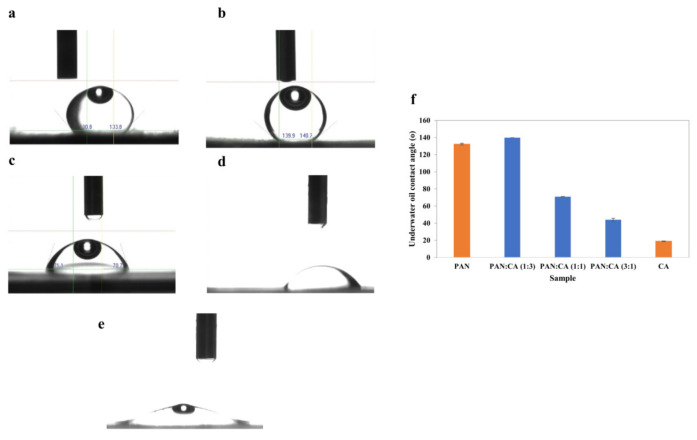
Images of underwater oil droplet on (**a**) PAN (**b**) 1:3 (**c**) 1:1 (**d**) 3:1 and (**e**) CA nanofibers and (**f**) underwater oil contact angle of PAN/CA nanofibers.

Figure 7 shows images of underwater oil droplets on PAN/CA nanofibers and the underwater oil contact angle of PAN/CA nanofibers. Nanofibers containing pure PAN were highly oleophobic with an underwater oil contact angle of 132.3°. In contrast, the underwater oil contact angle of pure CA nanofibers was around 18.95° (Figure 7e). On the other hand, the underwater oil contact angle decreased with an increase in the PAN:CA ratio from 1:3 to 3:1 in PAN/CA nanofibers (Figure 7). The maximum underwater oil contact angle was recorded for 1:3 PAN:CA ratio nanofibers with a value of 140°. The remarkable achievement of a high underwater oil contact angle in these nanofibers can be attributed to their surface’s hierarchical structure which consists of micro–nano-features. This structural arrangement causes the water to be retained within individual fibers, resulting in the forming of a triple-phase oil–water–solid interface. Subsequently, this configuration minimizes the contact area between oil and the surface, leading to the effective rolling-off of oil droplets from the nanofiber surface. In addition, introducing CA in the shell and PAN in the core of the nanofibers also revealed a super-hydrophilic nature due to the additional hydrophilic functionalities and similar underwater superoleophobic characteristics. The synthesized PAN/CA nanofibers with super-hydrophilic and underwater superoleophobic features are considered promising for oil–water separation. Accordingly, the optimal composite with a 1:3 PAN:CA ratio was selected for oil–water filtration experiments.

### 3.2. Performance of PAN/CA Nanofibers

#### Oil–Water Mixture Separation

**Figure 8:**
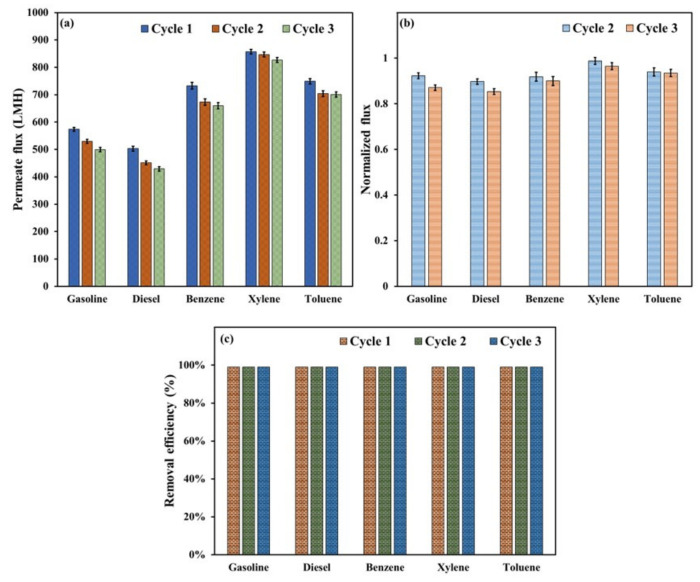
(**a**) Water permeate flux over three filtration cycles of different oil–water mixtures, (**b**) the normalized flux measurements of the 2nd and 3rd cycles, (**c**) removal efficiency of the oils for three consecutive filtration cycles.

The membranes’ performance was investigated by separating five different oils (gasoline, diesel, benzene, xylene, and toluene) from water. The hydrophilic membrane that showed the best hydrophilic–oleophobic properties, i.e., the membrane with a composition of 1:3 PAN to CA, was utilized for the separation experiments. Figure 8a illustrates the water permeate flux for the different oil–water mixtures over three filtration cycles. The permeate flux measurements under gravity conditions were between 429.2 and 857.2 L m^−2^ h^−1^ (LMH), implying that the synthesized membranes fall in the range of ultrafiltration and microfiltration membranes. It can be seen that the lowest flux measurements were obtained for the diesel/water mixture, and the maximum flux was obtained for the xylene/water mixture. The differences in flux measurements depend on many factors, such as the physical properties of the oil, including density and viscosity. Moreover, the chemical interaction between the oil droplets/particles and the surface of the membrane and the deposition rate of oil particles onto the membrane surface affect the permeate flux measurements. 

As visually noticed in the filtration experiments, the total filtration time of the diesel was relatively higher than that of other oils. The filtration time for 100 mL of mixture was in the following order: diesel > gasoline > benzene > toluene > xylene. The total filtration time was around 13 min for diesel, 10 min for gasoline, and 4–5 min for benzene, xylene, and toluene. These results suggested that the deposition of diesel particles onto the membrane surface was more evident than other types of oils. These conclusions can be supported by the decline in permeate flux with filtration cycle number (see Figure 8a). However, the reduction percentage throughout three filtration cycles for diesel was more noticeable; for instance, the permeate flux decreased from 502.9 LMH to 429.2 LMH and from 857.2 LMH to 826.9 LMH for diesel and xylene, respectively. This reduction represented 12.9% and 3.5% declines in permeate flux over the three cycles for diesel and xylene, respectively. Despite this reduction and regardless of the type of oil, the normalized flux values (see Figure 8b) for the second and third cycles demonstrated good stability and reusability of the prepared membrane. The normalized flux values were 0.85, 0.87, 0.90, 0.94, and 0.96 after three diesel, gasoline, benzene, toluene, and xylene filtration cycles, respectively. These measurements showed that the membrane can recover 85% to 96% of its initial permeate flux after three consecutive oil–water filtration cycles.

**Figure 9:**
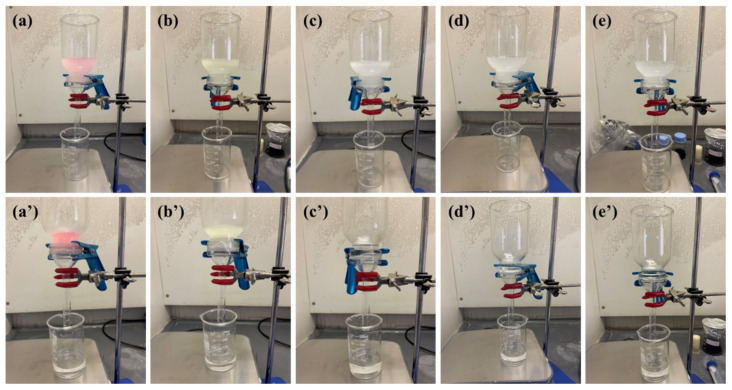
Images capturing the transformation of oil–water emulsions, both pre- and post-filtration, using the PAN/CA nanofiber membrane. Showing the emulsions of gasoline (**a**,**a’**), diesel (**b**,**b’**), benzene (**c**,**c’**), xylene (**d**,**d’**), and toluene (**e**,**e’**).

Figure 8c illustrates the removal efficiency of the utilized oils over three filtration cycles. The results revealed more than 99.9% rejection efficiency for all oils over the three filtration cycles. To validate the membrane’s reusability in oil–water separation, we conducted filtration experiments involving diesel and xylene for five cycles. The outcomes revealed a consistently high efficacy, with a rejection rate exceeding 99.9% for oil and only a slight reduction in flux measurements. These findings strongly indicate that the electrospun membrane with a 1:3 PAN-to-CA ratio can effectively facilitate oil–water separation for at least five cycles, underscoring the practicality and durability of the membrane for repeated applications. Figure 9 shows digital photographs of the oil–water suspension at the beginning of the filtration experiments and the final state of the permeate and rejected oil at the end of the tests. It is seen that the permeate has clear water without any signs of turbidity, indicating an almost complete rejection of the oils. Therefore, despite a slight decline in the membranes’ flux with each filtration cycle, they exhibited ideal reusability regarding oil rejection. 

Table 2 summarizes the removal efficiency and flux measurements of selected electrospun membranes. Our membranes included one of the best membranes in terms of oil rejection. The flux measurements of our membranes seem to be less than those of previously reported electrospun membranes; however, it should be noted that our experiments were driven by gravity, and in most of the reported literature, the filtration experiments were driven by vacuum or applied pressure. Accordingly, our results suggest a promising potential for utilizing PAN/CA electrospun membranes to treat oil spills in various water resources. 

The separation mechanism can be attributed to several factors. First is hydrophilic–oleophobic synergy: the electrospun membrane’s composition of PAN and CA generates a synergistic influence. The PAN provides hydrophilic characteristics, while the CA shell adds to the oleophobic nature of the membrane. This synergy allows the membrane to strongly repel oil due to oleophobicity while passing water due to its hydrophilic properties. This synergistic effect also identifies the membrane’s selectivity in passing water droplets and rejecting oil droplets, which can be considered a separation mechanism. As demonstrated in FESEM images, the fabricated membranes showed high porosity and massive permeability potential, which can be considered capillary forces that allow water droplets to flow within the membrane. The interfacial tension also plays a significant role in repelling oil droplets. The composite membrane’s super-hydrophilic and oleophobic properties reduce the interfacial contact between oil and the membrane, keeping the oil phase separated on the top surface of the membrane. 

## 4. Conclusions

In conclusion, hydrophilic–oleophobic PAN/CA nanofibers were successfully produced through one-step core–shell electrospinning. These nanofibers exhibit remarkable wettability properties, characterized by super-hydrophilicity and underwater superoleophobicity. This exceptional surface behavior makes them highly promising for efficient oil–water separation applications. The versatility of these nanofibers is evident as they effectively separate various oils from water, including gasoline, diesel, benzene, xylene, and toluene. The synthesized nanofibers are a reliable substrate, ensuring a high separation efficiency while maintaining stability and durability. The current work introduces a convenient fabrication method for nanofibers designed for on-demand oil–water separation, and these nanofibers show excellent reusability. Notably, a maximum separation efficiency of up to 99.9% and permeate flux values between 429.2 and 857.2 LMH make this technology valuable in scenarios where effective oil–water separation is crucial for environmental protection and safety.

## Figures and Tables

**Table 1:** Pumps’ flow rate of PAN and CA during electrospinning of PAN/CA nanofibers.

Sample	Pump 1 (PAN) Flow Rate (Core) (mL/h)	Pump 2 (CA) Flow Rate (Shell) (mL/h)
PAN	0.2	--
1:3	0.2	0.6
1:1	0.2	0.2
3:1	0.6	0.2
CA	--	0.2

**Table 2:** Summary of the performance of various electrospun membranes for oil–water separation.

Electrospun Membrane	Oil	Rejection Efficiency	Flux (LMH)	Ref.
Polyether sulfone/Fe_3_O_4_	Crude oil	94.0%	3200	[41]
PAN/Cellulose	n-hexane	97.3%	~1890	[2]
	Diesel	87.9%	~1500	
	Toluene	~95.0%	~1990	
PAN/s-kaolin	Bromobenzene	97.5%	1875	[42]
	Trichloromethane	98.0%	1800	
	Dichloromethane	98.0%	1950	
	Tetrachloromethane	96.5%	1780	
PAN/MWCNT-COOH/polyethyleneimine	n-hexane	84.0%	6013	[43]
	n heptane	84.0%	4175	
Poly(vinyl alcohol)/Agar	Toluene	98.3%	320	[44]
	Chloroform	99.2%	450	
	Diesel	97.2%	89.0	
PAN/GO/SiO_2_	Toluene	98.0%	460	[45]
CA/GO	Toluene	>99%	3650	[46]
	n-decane	>99%	3600	
	Hexane	>99%	3580	
PAN/Cellulose nanocrystals/polyethyleneimine/SiO_2_	Hexane	>99.4%	775	[47]
	Diesel	>99.4%	724	
	Toluene	93.0%	609	
	Petroleum ether	>99.4%	537	
PAN/CA	Diesel	>99.9%	502.9	This study
	Gasoline	>99.9%	573.6	
	Benzene	>99.9%	732.9	
	Toluene	>99.9%	748.9	
	Xylene	>99.9%	857.2	

## Data Availability

All data generated from this study are presented in the manuscript.
